# Supplementary material for: Correlation of Somatostatin Receptor 1–5 Expression, [68Ga]Ga-DOTANOC, [18F]F-FDG PET/CT and Clinical Outcome in a Prospective Cohort of Pancreatic Neuroendocrine Neoplasms
Source: Cancers (Basel). 2021 Dec 29;14(1):162. doi: 10.3390/cancers14010162 (PMC8750461; doi:10.3390/cancers14010162)
Supplement: Supplementary file 1 [file cancers-14-00162-s001.zip › cancers-1499289-supplementary.pdf]

Table S1. Association between [68Ga]Ga-DOTANOC PET/CT and SSTR2 expression assessed by both membranous and overall expression.

		SSTR2 Membranous Expression						SSTR2 Overall Score		
		score1	score2	score3	score4			score1	score2	score3
[68Ga]Ga-DOTANOC, p = 0.043	positive, n	0	3	10	9	[68Ga]Ga-DOTANOC, p = 0.043	positive, n	0	3	19
	negative, n	1	0	0	0		negative, n	1	0	0
[68Ga]Ga-DOTANOC SUVmax, p = 0.334	median	4.4	23.8	29.0	32.9	[68Ga]Ga-DOTANOC SUVmax, p = 0.236	median	4.4	23.8	29.1
	IQR	4.4	13.3–93.3 [a]	16.6–63.7	17.2–62.4		IQR	4.4	13.3–93.3 [a]	17.0–53.5
	n	1	3	10	9		n	1	3	19

[a]range, due to the inability to define interquartile range, IQR.
